# Developing a common data model approach for DISCOVER CKD: A retrospective, global cohort of real-world patients with chronic kidney disease

**DOI:** 10.1371/journal.pone.0274131

**Published:** 2022-09-29

**Authors:** Supriya Kumar, Matthew Arnold, Glen James, Rema Padman

**Affiliations:** 1 Real World Evidence Data and Analytics, BioPharmaceuticals Medical, AstraZeneca, Gaithersburg, MD, United States of America; 2 Real World Evidence Data and Analytics, BioPharmaceuticals Medical, AstraZeneca, Cambridge, United Kingdom; 3 Formerly Cardiovascular, Renal, Metabolism & Epidemiology, BioPharmaceuticals Medical, AstraZeneca, Cambridge, United Kingdom; 4 Heinz College of Information Systems and Public Policy, Carnegie Mellon University, Pittsburgh, PA, United States of America; Mahidol University, Faculty of Tropical Medicine, THAILAND

## Abstract

**Objectives:**

To describe a flexible common data model (CDM) approach that can be efficiently tailored to study-specific needs to facilitate pooled patient-level analysis and aggregated/meta-analysis of routinely collected retrospective patient data from disparate data sources; and to detail the application of this CDM approach to the DISCOVER CKD retrospective cohort, a longitudinal database of routinely collected (secondary) patient data of individuals with chronic kidney disease (CKD).

**Methods:**

The flexible CDM approach incorporated three independent, exchangeable components that preceded data mapping and data model implementation: (1) standardized code lists (unifying medical events from different coding systems); (2) laboratory unit harmonization tables; and (3) base cohort definitions. Events between different coding vocabularies were not mapped code-to-code; for each data source, code lists of labels were curated at the entity/event level. A study team of epidemiologists, clinicians, informaticists, and data scientists were included within the validation of each component.

**Results:**

Applying the CDM to the DISCOVER CKD retrospective cohort, secondary data from 1,857,593 patients with CKD were harmonized from five data sources, across three countries, into a discrete database for rapid real-world evidence generation.

**Conclusions:**

This flexible CDM approach facilitates evidence generation from real-world data within the DISCOVER CKD retrospective cohort, providing novel insights into the epidemiology of CKD that may expedite improvements in diagnosis, prognosis, early intervention, and disease management. The adaptable architecture of this CDM approach ensures scalable, fast, and efficient application within other therapy areas to facilitate the combined analysis of different types of secondary data from multiple, heterogeneous sources.

## Introduction

Chronic kidney disease (CKD), characterized by a gradual loss of kidney function over time, is a major cause of morbidity, loss of quality of life, and death globally, with a prevalence of approximately 1 in 10 people that is increasing as the prevalence of associated conditions such as diabetes mellitus, cardiovascular disease, and hypertension also continue to grow [1]. Prevalence of complications such as anemia and hyperkalemia increase with the severity of CKD and can be life threatening, necessitating costly long-term pharmacological interventions to improve outcomes [2–5].

Developing initiatives to improve outcomes of patients with CKD and associated comorbidities and complications has been identified as a global priority [1, 6–11]. The DISCOVER CKD program is a unique, large-scale, multinational, longitudinal study of patients with a specific multimorbid health condition (CKD), comprising real-world data (RWD) including independent prospective and retrospective patient cohorts [12]. Through the generation of primary and secondary RWD, the DISCOVER CKD program aims to provide novel insights into the epidemiology of CKD, describing aspects including patient characteristics, disease progression, clinical outcomes, the patient journey–including comorbidity and pharmacotherapy burden, practice patterns, and clinical management of CKD [13].

The retrospective cohort of DISCOVER CKD comprises secondary data from >1.8 million patients, extracted from several established, anonymized electronic health record and claims databases that AstraZeneca has licensed for internal analysis or through external collaborations. Databases included at time of development are listed in **Table 1**, with the addition of more data sources planned. Data extracted include patient demographics, prescriptions, procedures, healthcare resource utilization and encounters, medical history, and laboratory values. A comprehensive list of all variables (>170) and detailed study objectives of DISCOVER CKD have been reported previously [12].

**Table 1:** Established databases included in DISCOVER CKD.

Status (CDM)	Database name	Country	Database type	Coverage	Reference
Included in CDM for 2020 analyses	TriNetX [14]	USA	EHR	Inpatient and outpatient	Topaloglu U, et al. *JCO Clin Cancer Informatics* 2018;2:1–10.
	Explorys (LCED) [15]	USA	EHR and claims	Inpatient and outpatient	https://www.ibm.com/watson-health/about/explorys
	DOPPS [16]	USA	EHR	Hemodialysis	Pisoni RL, et al. *Am J Kidney Dis* 2004;44:7.
	CPRD [17]	UK	EHR	Primary care, inpatient and outpatient, ER	Herrett E, et al. *Int J Epidemiol* 2015;44:827–836.
	JMDV [18]	Japan	EHR and claims	Inpatient and outpatient	Tanaka S, et al. *J Pharm Heal Care Sci* 2015;1:16.
Upcoming (2021)	J-CKD DB (Kawasaki Medical School) [19]	Japan	EHR	Inpatient and outpatient	Nakagawa N, et al. *Sci Rep* 2020;10:7351.

CDM, common data model; CPRD, Clinical Practice Research Datalink; DOPPS, Dialysis Outcomes and Practice Patterns Study; EHR, electronic health record; ER, emergency room; J-CKD DB, Japan Chronic Kidney Disease Database; JMDV, Japan Medical Data Vision; LCED, Limited Claims and Electronic Health Record Database.

To facilitate studies including multiple diverse data sources, many RWD analyses utilize a common data model to standardize terminologies [20]. Typically, between sources of RWD, medical events are recorded in many different coding systems (for example, International Classification of Diseases [ICD]-9, ICD-10, UK Read Code) which necessitates standardization. In addition, differences, such as those in settings, and in regional and local clinical practices [21], may result in heterogeneity in reporting of laboratory units, which requires extensive pre-work to ensure that data are standardized before being consolidated. In terms of the multiple secondary datasets included for analysis within the DISCOVER CKD retrospective cohort, data structure and coding systems were disparate, from different settings and populations, and required consolidation into a discrete and standardized database. Pre-work was also necessary to consolidate the varying clinically acceptable definitions of patients with CKD from within each source database, to identify patients meeting inclusion/exclusion criteria outlined in the DISCOVER CKD study protocol (detailed previously) [12]. As such, the DISCOVER CKD program required a common data model (CDM) approach.

CDMs create common value sets to standardize disparate data structures, support scalability, streamline multi-database analysis, and enhance data interoperability. The intended result is the generation of robust real-world evidence (RWE) [20]. CDMs for the harmonization of healthcare data have been developed previously; notable industry standard examples include Informatics for Integrating Biology and the Bedside (i2b2) [22], The Observational Outcomes Medical Partnership (OMOP) CDM, managed by Observational Health Data Sciences and Informatics (OHDSI) [23], Sentinel, launched by the US Food and Drug Administration (FDA) [24, 25], and the Patient-Centered Outcomes Research Network (PCORnet) [26, 27].

Industry standard CDMs, such as the OMOP CDM, have been used previously to combine databases for observational studies. They are typically used to convert the entire data source population through the mapping of all events in a given data source code-to-code. However, in DISCOVER CKD, the volume of planned studies posed an opportunity to create a template for study-specific analysis datasets, serving as a study-specific CDM or harmonized analytical dataset. The use of the CDM described in this paper is specific to the DISCOVER CKD study and complementary to the source data format. The source dataset can be OMOP format or other industry standard formats, and the study-specific CDM in this paper would harmonize the medical events to the study semantics, improving efficiency of the data scientists’ study initiation workflow.

### Objectives

The objective of this report is to describe the development of a flexible CDM that could achieve the following goals:

- Facilitate pooled, patient-level analysis (where possible) in addition to aggregated/meta-analysis of a multinational, retrospective (secondary), longitudinal database of routinely collected patient data from disparate data sources, to support the realization of DISCOVER CKD research objectives.
- Achieve core user needs of ease of use, speed, and scalability through the generation of a single, harmonized data source that increases workflow efficiency.
- Be effectively refreshed, adapted, updated, and expanded with new data sources in a streamlined and well-documented manner, ensuring applicability for future use within DISCOVER CKD and other therapy areas.

## Materials and methods

### Development of a flexible CDM approach

**Fig 1:**
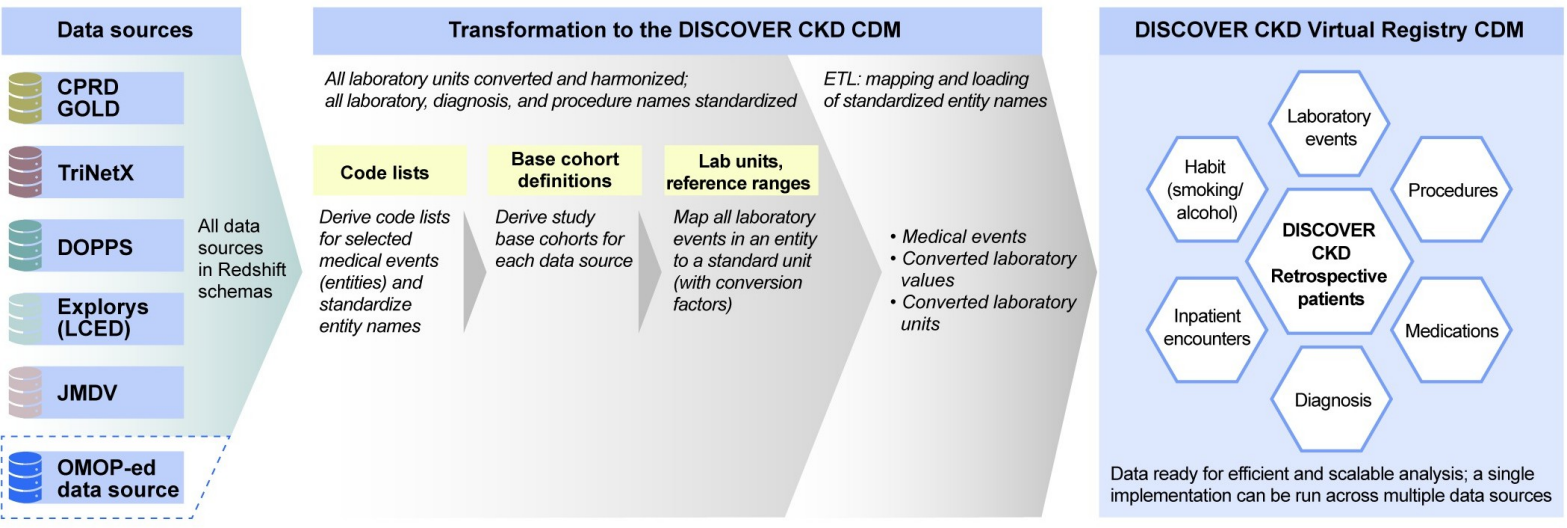
DISCOVER CKD CDM architecture. Blue dashed line denotes potential data sources that could be added to the flexible CDM. CDM, common data model; CKD, chronic kidney disease; CPRD, Clinical Practice Research Datalink; DOPPS, Dialysis Outcomes and Practice Patterns Study; ETL, extract, transform, load; JMDV, Japan Medical Data Vision; LCED, Limited Claims and Electronic Health Record Dataset; OMOP, Observational Outcomes Medical Partnership.

**Fig 2:**
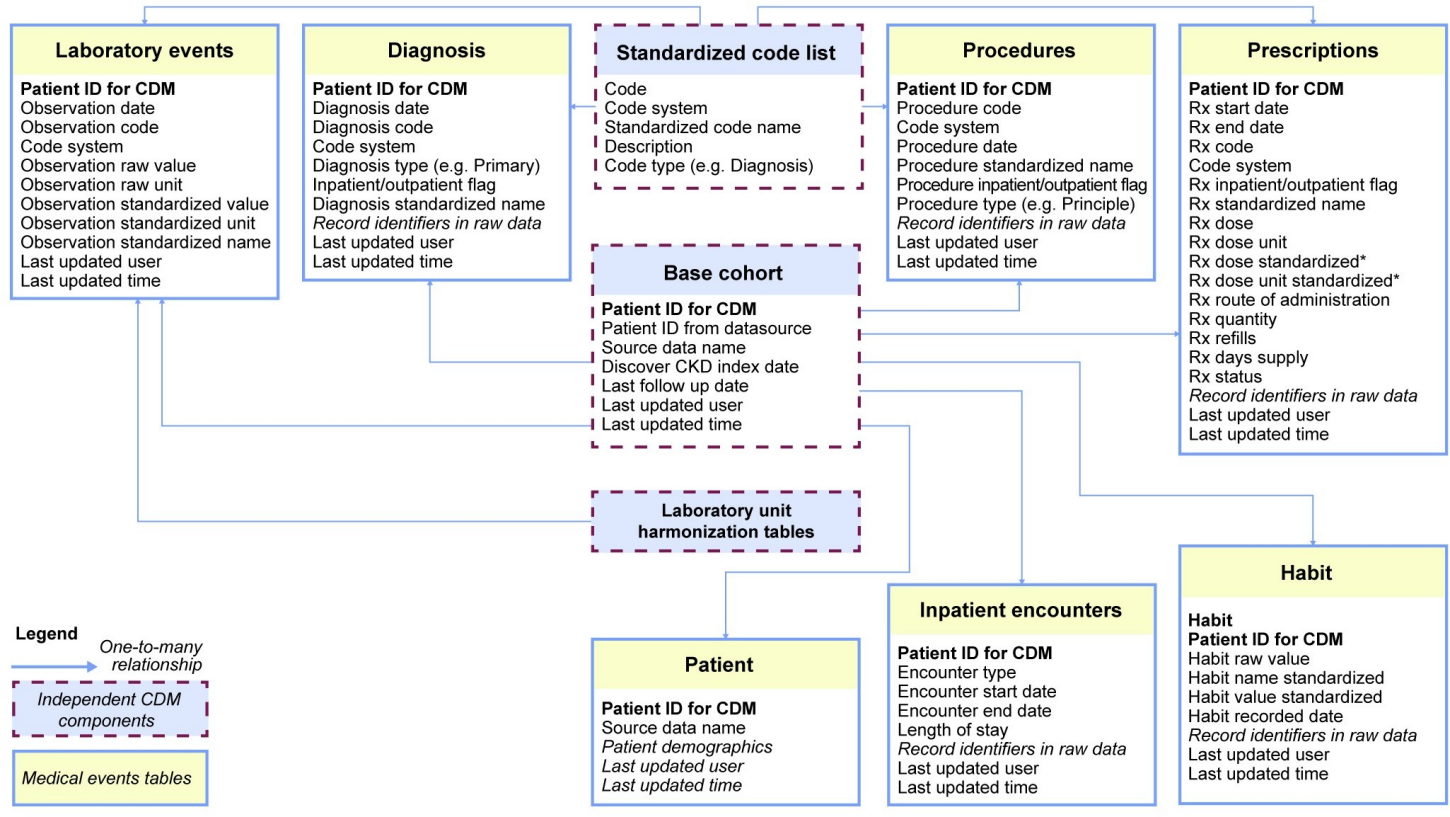
Simplified entity relationship diagram. *Prescription dose and dose standardization are pending. CDM, common data model; CKD, chronic kidney disease; ID, identification; Rx, prescription.

Three key components of the flexible CDM were designed to function in an independent manner, to address the needs of the DISCOVER CKD program, and to be easily updated for future application to other therapy areas: (1) standardized code lists (to unify medical events from different coding systems); (2) laboratory unit harmonization tables (comprising conversion factors to standardize laboratory units); and (3) base cohort definitions (selection criteria to define source databases patients for inclusion; **Fig 1**). These components were developed as separate entities to be easily replaced or extended as required for novel applications (**Fig 2**). For example, base cohorts could easily be replaced, and new codes or standardized terms (medical events/encounters) could be added to the code lists. Once completed, extract, transform, load (ETL) logic, coding, and procedures were applied to the components to initiate the data model. The ETL process remained constant per data source, while the three independent components were exchanged.

### Development of standardized code lists

The DISCOVER CKD protocol comprised >150 entity types, spanning diagnosis, prescriptions, procedures, and laboratory values, requiring code lists across different coding systems. The target data standards for DISCOVER CKD are summarized in **S1 Table**. Relevant code lists were generated by the study team (**S2 Table**) and externally validated by a clinical coder. Coding systems were searched for terms related to each entity and irrelevant codes were excluded. Clinicians on the study team verified the final code lists in an additional confirmation step, with any discrepancies resolved through cross-functional discussion and consensus. Following verification, code lists were loaded into a single database table (**S3 Table**), hereon referred to as the reference code lists table. A singular reference table of code lists ensured that scripts loaded into the data model could be kept as simple as possible. Code lists were harmonized for all entity types included in the protocol, across all data sources, to ensure studies on the CDM had reliable standardized entity names for analysis. For example, entity codes for type 2 diabetes—ICD-10 code E11.34 and Read code C100112—were both labeled as ‘type 2 diabetes’.

One-to-one mapping was not performed as it was not possible to reach complete harmonization between different coding systems. All medical events were standardized at the entity level, while interpretation of events was undertaken at analysis. Contextual considerations by data source and country were required. For example, CKD hospitalization events had to be interpreted with consideration to country-specific differences; in Japan, a patient could be hospitalized to test for CKD, resulting in a CKD hospitalization event which was not due to CKD.

### Harmonization of laboratory units

The DISCOVER CKD retrospective patient cohort includes data on several biochemical laboratory measures commonly collected in clinical practice. Different reporting standards between data sources (such as the laboratories in hospitals, practices, or clinics) resulted in differing laboratory units. For example, data sources showed up to six different units for creatinine: μmol/L, mmol/L, mol/L, microU/L, mg/dL, mmol, mg/L, and g/L.

**Fig 3:**
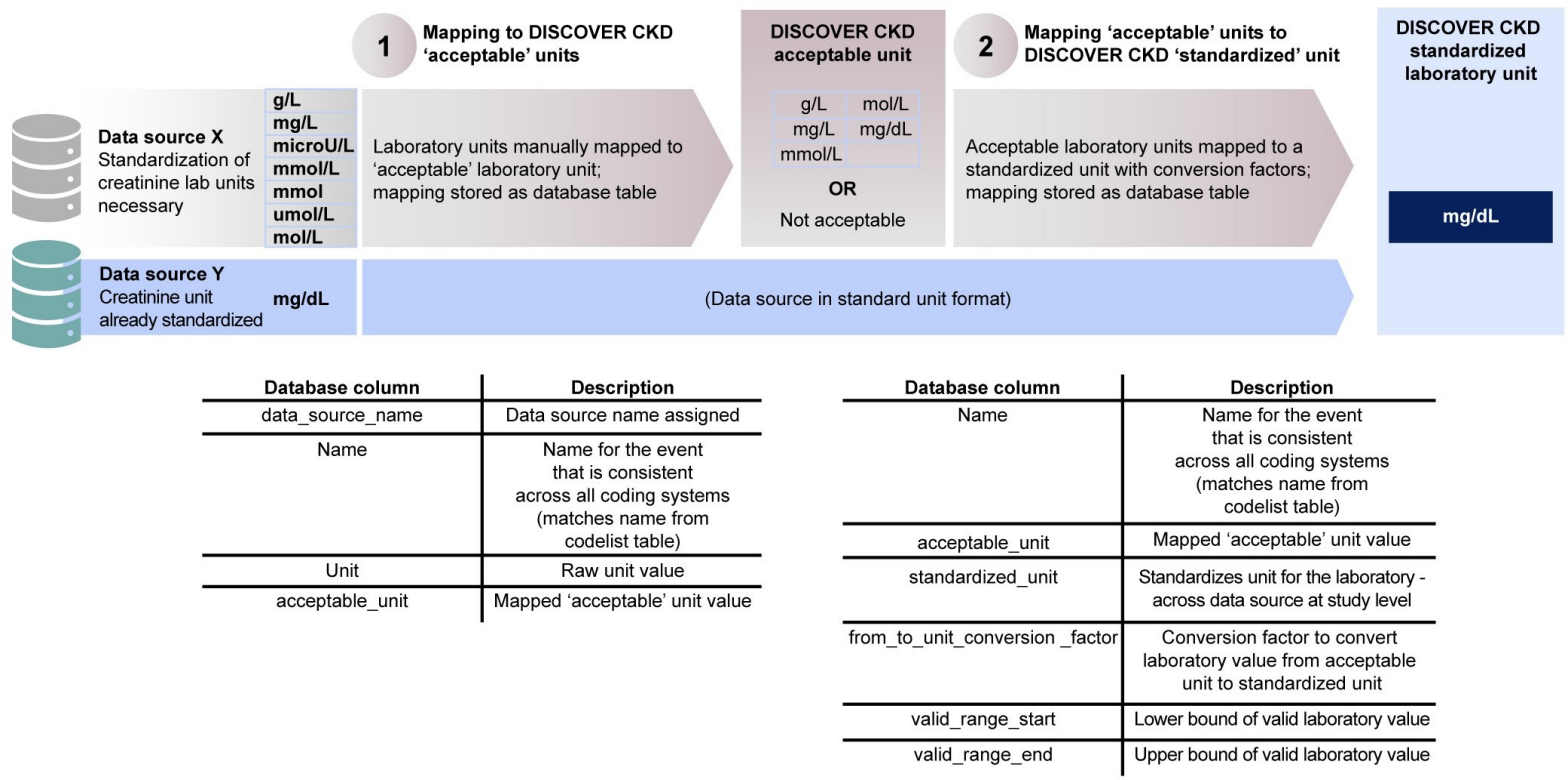
Example laboratory unit harmonization: Creatinine units. Acceptable units were defined as any unit that was valid for the laboratory and could be converted to the standardized unit. CKD, chronic kidney disease.

Standard units for each laboratory measure, to be reported in DISCOVER CKD studies and analyses, were selected and confirmed within the study team. Clinically plausible lower and upper range values for each laboratory measure were also confirmed with medical input to ensure that implausible laboratory values were not included. Units for each laboratory were harmonized using relevant conversion factors [28, 29]. Implementation of standardization for each data source, and for each laboratory unit, required two principal steps (**Fig 3**): (1) manual mapping to DISCOVER CKD acceptable units; and (2) conversion to DISCOVER CKD standardized units. Acceptable units were defined as any unit that was valid for the laboratory measure and could be converted to the standardized unit. For data sources containing significant laboratory records with an unknown unit, where possible, frequency distributions were used to map and assign the closest logical unit matching the distribution of laboratory values. For example, if hemoglobin values were given without a known unit, but values within the data source ranged from 3 to 22 with an appropriate distribution, the unit g/dL was applied. Relevant conversion factors [28, 29] for each laboratory unit were loaded as a reference table. Using programmed conversion factors, the acceptable unit, across data sources, was converted to the standardized unit as part of the data-load to the CDM. The final laboratory table in the CDM maintains both the raw unit and value, and the standardized unit and value.

### Base cohort definition

The standardized code list table was utilized to derive the DISCOVER CKD base cohort for each data source. Base cohorts were stored in separate tables per data source, where each data source was given a unique name (stored in a variable called data_source_name). Base cohorts were then combined to create a cohort of all patients with CKD in DISCOVER CKD in one table, in line with data governance policies applying to each data source. The two-step process of creating data source-specific base cohorts prior to combination into a cross-data source base cohort was adopted to ensure that individual data sources could be refreshed with new data, without the need to refresh base cohorts of all data sources.

Briefly, patients with CKD were identified within each database for the DISCOVER CKD base cohort by meeting any of three criteria: (1) documented diagnostic code (e.g. ICD-10) for CKD stages 3A through to kidney failure; (2) two estimated glomerular filtration rate (eGFR) measures [30] of <75 mL/min/1.73 m^2^ recorded >90 days apart (maximum, 730 days) from January 1, 2008; or (3) a code for chronic (duration, >30 days) renal replacement therapy (hemodialysis and peritoneal dialysis). The CKD Epidemiology Collaboration (CKD-EPI) equation without race was used to calculate eGFR; for Japanese data, eGFR was calculated from serum creatinine using the revised equations by Matsuo et al. (2009) modified for the standard physique [12, 31]. Once a study-specific cohort is identified, data can be imported into any statistical software for analysis.

### Data mapping and data model implementation

Standardized medical entities are held in seven core tables outlined in **Fig 2**, with an additional table for patient demographics. Mapping between the source data and the CDM was documented for each data source. Source columns and tables, the target column and table, and the logic used to transform the source entity to the entity in the CDM were documented at a column level to enhance traceability and quality control. Harmonization of entity descriptors, such as diagnosis type or inpatient/outpatient events in the relevant core tables, was included as a pertinent document step for the maintenance and extension of the CDM. ‘Record identifier’ and ‘last updated information’ columns were included in the target (CDM) core tables for traceability. Record identifiers, such as encounter ID and record ID associated with the medical entity, were retained in the CDM to enable matching of an entity in the CDM to the respective entity in the source data. This enables easy extension of the CDM to include data points specific to the data source that are currently not included, such as cost. These columns were also key for data quality checks during study implementation. Last updated information comprised two columns to record which user performed the upload and when the medical entity was loaded into the CDM.

Once the mapping document was complete, technical validation was undertaken by data scientists who had a working knowledge of the data sources prior to implementation of ETL logic to initiate the data model. The ETL process outlines the database functions implemented in the following steps: taking data from the original source (‘extraction’); standardizing disparate datasets using the CDM components described above (‘transformation’); and loading into the end database (to comprise the DISCOVER CKD retrospective cohort; ‘loading’). Face validity of the data output was confirmed systematically by the study team once all data were loaded to ensure the pooled baseline covariates of patients with CKD were reasonable and in line with existing literature. These validation steps ensure that the DISCOVER CKD secondary data output is of high quality and suitable for analyses.

### Data management and procedures

Datasets were stored in the Amazon Redshift cloud data warehouse. Python Jupyter Notebooks were used to maintain the readability of all documented steps and initiated Structured Query Language (SQL) scripts. Papermill (Python) was utilized to execute notebooks and SQL scripts, and to organize the steps to build, refresh, and add new data sources [32]. Code was maintained on an internal Bitbucket repository. ETL logic to initiate the data model was implemented in SQL.

All data in the flexible CDM were loaded per local data requirements. For Dialysis Outcomes and Practice Patterns Study (DOPPS), AstraZeneca licensed a deidentified limited data set from Arbor Research Collaborative for Health (Michigan, USA) pursuant to a data use and licensing agreement between the parties.

### Ethics approval

The DISCOVER CKD master protocol and statistical analysis plan were reviewed by the AstraZeneca team and collaborating scientific committee, and submitted for review and approval to AstraZeneca’s internal governance. Extraction of clinical data was conducted in accordance with country-specific data privacy laws, governance, ethical approvals (where applicable), and patient consent (where applicable); only anonymized data were utilized for DISCOVER CKD. For the UK Clinical Practice Research Datalink (CPRD), the DISCOVER CKD protocol was approved by an Independent Scientific Advisory Committee (ISAC protocol number: 19_226A4). Institutional Review Board approval was not required as the study did not involve the collection, use, or transmittal of individual identifiable data.

## Results

### Secondary data and analyses in DISCOVER CKD: 2020 analytical output

At the time of development of the DISCOVER CKD retrospective cohort, the CDM approach was used to harmonize secondary data from 1,857,593 patients with CKD from five data sources across three countries (UK CPRD, US DOPPS, US Limited Claims and Electronic Health Record Dataset [LCED] 2019, US TriNetX, and Japan Medical Data Vision) into a discrete database for RWE generation (**Table 1; S1 Fig**). DOPPS has subsequently been removed from DISCOVER CKD future analyses in line with data use and licensing agreements; other secondary datasets from DISCOVER CKD are planned for future inclusion in the CDM.

Standardized secondary data metrics, and the resultant baseline covariates of the population included in DISCOVER CKD, overall and by database, are summarized in **Table 2**. Due to data privacy restrictions, data derived from LCED could not be pooled with data from other databases for analysis of the overall DISCOVER CKD cohort.

**Table 2:** Secondary data and baseline covariates of patients included in DISCOVER CKD.

	Overall	TriNetX	Explorys (LCED)	DOPPS	CPRD	JMDV
**Secondary data**						
**Database size,^a^ GB**	76.8	53.1	16.9	7.1	15.4	8.1
**Total records, n**	1,042,035,868^b^	417,339,825	181,577,200	3,852,045	250,747,268	46,344,726
**Procedure events**	22,756,095^b^	10,962,854	5,129,753	16,626	3,512,078	4,143,808
**Coded outcomes, n**	21	20	19	1	18	14
**Diagnosis events**	168,815,184	84,116,926	73,572,637	108,890	7,865,001	3,151,730
**Coded outcomes, n**	63	57	57	18	60	56
**Drug prescription events**	364,318,203^b^	46,335,922	25,870,962	452,163	124,011,918	23,449,842
**Medication classes, n**	57	42	46	20	46	38
**Laboratory/biochemistry measurements**	458,706,628	271,238,863	68,540,692	3,209,992	102,051,746	15,104,149
**Classes, n**	44	31	33	21	35	19
**Baseline covariates^c^**						
**Mean age at index, years (SD)**	68.5 (13.69)	65.87 (13.41)	69.93 (13.98)	-	72.03 (12.76)	75.99 (12.76)
**Female, n (%)**	417,512 (55.7)	264,633 (56.3)	49,845 (54.7)	8109 (42.7)	104,650 (58.0)	40,120 (50.0)
**Mean BMI, kg/m^2^ (SD)**	28.89 (5.67)	28.88 (5.37)	30.19 (7.24)	-	28.91 (6.26)	-
**Mean baseline eGFR, mL/min/1.73 m^2^ (SD)**	49.45 (15.56)	48.3 (16.48)	52.07 (14.75)	-	53.7 (11.08)	49.16 (14.76)
**CKD stage 2 or functional decline, n (%)**	139,973 (18.7)	99,497 (21.2)	31,458 (34.6)	-	4683 (2.6)	35,793 (44.6)
**CKD stages 3–5, n (%)**	273,092 (36.4)	13,4612 (28.6)	49,579 (54.5)	18,982 (100.0)	80,650 (44.7)	38,848 (48.4)
**Diabetes mellitus, type 2, n (%)**	224,656 (30.0)	162,293 (34.5)	36,232 (39.8)	18 (0.1)	33,771 (18.7)	28,574 (35.6)
**Hypertension, n (%)**	483,061 (64.4)	311,769 (66.3)	76,185 (83.7)	15,058 (79.3)	98,554 (54.6)	57,680 (71.9)
**Heart failure, n (%)**	138,400 (18.5)	71,987 (15.3)	18,287 (20.1)	4588 (24.2)	12,528 (6.9)	49,297 (61.5)

^a^Overall database size is smaller than the sum of contributing sources due to data compression inside the Amazon Redshift cloud data warehouse.

^b^Overall events contain additional composite events generated following harmonization of data sources into the DISCOVER CKD retrospective cohort.

^c^Overall baseline covariates exclude LCED, which could not be pooled due to data privacy restrictions.

BMI, body mass index; CKD, chronic kidney disease; CPRD, Clinical Practice Research Datalink; DOPPS, Dialysis Outcomes and Practice Patterns Study; eGFR, estimated glomerular filtration rate; JMDV, Japan Medical Data Vision; LCED, Limited Claims and Electronic Health Record Dataset; SD, standard deviation.

The flexible CDM was developed in 2020 and, in the same year, data from pooled and meta-analyses of DISCOVER CKD were disseminated in 12 poster presentations at global congresses, with a further eight poster presentations in 2021. Several manuscripts are currently under development based on secondary data derived from this patient cohort, covering CKD and complications including anemia and hyperkalemia across CKD subgroups in multiple countries.

## Discussion

RWE generated from routinely collected data is increasingly being sought by decision‐makers. Creating datasets suitable for the generation of RWE from longitudinal healthcare databases involves extensive data processing; reporting the study parameters utilized, such as the methods and decisions undertaken in the CDM approach, can improve reproducibility, rigor, and confidence in the RWE generated from such databases [33, 34]. The flexible CDM approach described here was developed initially for the implementation and analysis of a closed cohort specific to the needs of, and criteria for, the retrospective cohort of the DISCOVER CKD program, for large-scale analyses of patients with CKD where results are obtained more rapidly than with the conventional approach of working on each study independently. The rapid generation of diverse, extensive RWE from patients with CKD from the DISCOVER CKD program throughout 2020 and onwards has only been possible using such an approach.

Incorporating the principles of speed, ease of use, adaptability, and scalability, this flexible CDM approach may provide a more agile harmonization of secondary data than other existing CDMs could offer. Adaptability and scalability were achieved by designing CDM components to function independently of one another. Storing individual data sources separately in a federated approach (i.e. a unified approach to base cohort tables) streamlines future modifications to the CDM, as users need only refresh, replace, or update the components of interest. For example, if a data license is due to expire, it is possible for the user to remove specific data from the refresh without impacting other data in the CDM, as demonstrated with the removal of DOPPS from future analyses of the DISCOVER CKD retrospective cohort.

The quality of the curation of underlying data is a consideration when determining the value of RWE derived from a CDM [20]. To achieve high-quality RWE from the given output, CDM development requires expertise within several areas: significant knowledge of the data, understanding of epidemiologic study design, and competency within RWE analysis principles [20]. Through the inclusion of epidemiologists, clinicians, informaticists, and data scientists within validation steps throughout CDM development, analyses derived from this flexible CDM approach should prove to be of high quality, sufficient for the generation of RWE from the DISCOVER CKD retrospective cohort.

There were some challenges in the development of this CDM approach. The time and effort required to create and refine correct code lists, and to obtain permission from data owners to conduct this study, were considerable. Establishing a setup in which updates to code lists could occur during study implementation, without detriment to the study, was key. Code was written with the assumption that it would need to be rerun, and efforts were made to facilitate rerunning and reloading the model, such that this demanded minimal effort from the programmer or data scientist.

This CDM approach offers a solution to increase RWE insights through the DISCOVER CKD program, integrating data from >1.8 million patients with CKD for analysis. Novel insights gleaned from real-world, routine-care data on epidemiology, patient burden, practice patterns, and patient outcomes [35–38] can help to improve the understanding of CKD, and DISCOVER CKD may therefore facilitate improvements in diagnosis, prognosis, early intervention, and disease management. In addition, implementation of an effective, transparent, and adaptable CDM approach can maximize the evidence gained from RWD.

### Future work

The approach to building a CDM as described here may act as a standard to help improve scale and efficiency of analyses within future studies, by harmonizing different data sources in future multi-database studies (across various therapeutic areas) or by facilitating the build of disease-specific secondary registries available for *ad hoc* analysis and studies. Furthermore, as the value of RWE is increasingly being recognized by regulatory bodies worldwide, such as the United States FDA [39] and UK Medicines & Healthcare products Regulatory Agency (MHRA) [40], such evidence may contribute to future regulatory decision making.

### Strengths

There are several strengths of the flexible CDM described in this study, and relating to the application of this CDM for analysis of the DISCOVER CKD retrospective cohort. The measures described above detail an approach to building a CDM suitable for adaptation for use within other therapy areas (allowing other groups to answer important research questions), and that is scalable such that it can accommodate new data sources and provide coverage of additional types of data (for example, costs), allowing a longevity of impact and evidence generation that may not have been achieved otherwise. These measures will also facilitate the application of this CDM approach to other therapy areas with fewer pre-work requirements.

In addition, novel RWD types (such as those derived from sensors and devices used for patient-reported outcomes) could easily be incorporated into the CDM approach at a level suitable to the study. For example, novel RWD types could be added as a new entity and loaded into an existing table (i.e. ‘observations’), or a new table could be added to the CDM. Although the current version of the CDM houses data in one place, the federated approach to data management ensures flexibility around restrictive data licensing policies; data sources can be converted to CDM format separately whilst remaining in the source location [41]. Likewise, individual participant data could be stored across multiple locations, and the CDM could be adapted for each data source and applied *in situ*, with analyses designed and run separately or in parallel as required.

Finally, data loss may be less common within this built-for-purpose CDM approach compared with industry standard CDMs that map vocabularies code-to-code. Each data source has its own curated code list of labels at the entity/event level; specific events in one coding standard (i.e. ICD-10) are not linked to those in another standard (i.e. Read code) approach. There was no data loss in medical diagnosis, procedures, and prescription events; only laboratory events with values that fell outside the acceptable range, or units that could not be verified, were excluded. Exclusion of data was limited to <1% of laboratory data, which comprised invalid values without units.

### Limitations

The approach described here also has some limitations. Although this CDM approach has been designed for adaptation to other therapy areas, the transformation rules applied to laboratory value conversions have been developed for use within the CKD therapy area and may not be appropriate for all circumstances. Collaboration with epidemiologists, clinicians, informaticists, and data scientists with specific knowledge of the therapy area is essential. Some databases did not allow for pooling of data due to data privacy restrictions. Although sufficient-quality RWE can inform decision making, RWD, including that incorporated into the CDM for DISCOVER CKD, has inherent limitations that have previously been described in detail.

## Conclusions

The flexible CDM approach used for the DISCOVER CKD study provides a scalable, fast, and efficient platform to harmonize secondary data from multiple heterogeneous sources, facilitating analysis of a large retrospective cohort of patients to provide novel insights into the epidemiology of CKD by describing real-world patient characteristics, disease progression, clinical outcomes, the patient journey, practice patterns, and clinical management of patients with CKD. Furthermore, owing to its flexible and adaptable architecture, this CDM approach may be applied to other therapy areas to facilitate the combined analysis of different types of secondary data.

## Supporting information

S1 Fig DISCOVER CKD common data model overview. (TIF)

S1 Table Target data standards for DISCOVER CKD. (DOCX)

S2 Table Study team members, roles, and affiliations. (DOCX)

S3 Table Reference table of code lists. (XLSX)

## References

[1] Lv JC, Zhang LX. Prevalence and Disease Burden of Chronic Kidney Disease. In: Liu B-C, Lan H-Y, Lv L-L, editors. Renal Fibrosis: Mechanisms and Therapies. Singapore: Springer Singapore; 2019. p. 3–15. 10.1007/978-981-13-8871-2_1 31399958

[2] Ryu SR, Park SK, Jung JY, Kim YH, Oh YK, Yoo TH, et al. The Prevalence and Management of Anemia in Chronic Kidney Disease Patients: Result from the KoreaN Cohort Study for Outcomes in Patients With Chronic Kidney Disease (KNOW-CKD). J Korean Med Sci 2017;32:249–56. doi: 10.3346/jkms.2017.32.2.249 ; PubMed Central PMCID: PMC5219990. 28049235 PMC5219990

[3] Toft G, Heide-Jorgensen U, van Haalen H, James G, Hedman K, Birn H, et al. Anemia and clinical outcomes in patients with non-dialysis dependent or dialysis dependent severe chronic kidney disease: a Danish population-based study. Journal of Nephrology 2020;33:147–56. doi: 10.1007/s40620-019-00652-9 ; PubMed Central PMCID: PMC7007417. 31587136 PMC7007417

[4] Kovesdy CP, Regidor DL, Mehrotra R, Jing J, McAllister CJ, Greenland S, et al. Serum and dialysate potassium concentrations and survival in hemodialysis patients. Clin J Am Soc Nephrol 2007;2:999–1007. doi: 10.2215/CJN.04451206 . 17702709

[5] Karaboyas A, Zee J, Brunelli SM, Usvyat LA, Weiner DE, Maddux FW, et al. Dialysate Potassium, Serum Potassium, Mortality, and Arrhythmia Events in Hemodialysis: Results From the Dialysis Outcomes and Practice Patterns Study (DOPPS). Am J Kidney Dis 2017;69:266–77. doi: 10.1053/j.ajkd.2016.09.015 ; PubMed Central PMCID: PMC5520979. 27866964 PMC5520979

[6] Cockwell P, Fisher LA. The global burden of chronic kidney disease. Lancet 2020;395:662–4. doi: 10.1016/S0140-6736(19)32977-0 . 32061314

[7] GBD Chronic Kidney Disease Collaboration. Global, regional, and national burden of chronic kidney disease, 1990–2017: a systematic analysis for the Global Burden of Disease Study 2017. Lancet 2020;395:709–33. doi: 10.1016/S0140-6736(20)30045-3 ; PubMed Central PMCID: PMC7049905. 32061315 PMC7049905

[8] Burckhardt P, Nagin D, Padman R. Multi-Trajectory Models of Chronic Kidney Disease Progression. AMIA Annu Symp Proc 2017;2016:1737–46. PubMed Central PMCID: PMC5333229. 28269932 PMC5333229

[9] Zhang Y, Padman R. Innovations in chronic care delivery using data-driven clinical pathways. Am J Manag Care 2015;21:e661–8. 26760429

[10] Zhang Y, Padman R, Patel N. Paving the COWpath: Learning and visualizing clinical pathways from electronic health record data. J Biomed Inform 2015;58:186–97. doi: 10.1016/j.jbi.2015.09.009 . 26419864

[11] Chen TK, Knicely DH, Grams ME. Chronic Kidney Disease Diagnosis and Management: A Review. JAMA 2019;322:1294–304. doi: 10.1001/jama.2019.14745 ; PubMed Central PMCID: PMC7015670. 31573641 PMC7015670

[12] Pecoits-Filho R, James G, Carrero JJ, Wittbrodt E, Fishbane S, Sultan AA, et al. Methods and rationale of the DISCOVER CKD global observational study. Clinical Kidney Journal 2021;14:1570–8. doi: 10.1093/ckj/sfab046 ; PubMed Central PMCID: PMC8264307. 34249352 PMC8264307

[13] AstraZeneca. NCT04034992: A Study on Patients With Chronic Kidney Disease (CKD) to Assess Treatment Experience and Patterns, Effect of the Treatment, Patient Outcomes and Patient Quality of Life 2019 [cited 2021 January 29]. Available from: https://clinicaltrials.gov/ct2/show/NCT04034992.

[14] Topaloglu U, Palchuk MB. Using a Federated Network of Real-World Data to Optimize Clinical Trials Operations. JCO Clin Cancer Inform 2018;2:1–10. doi: 10.1200/CCI.17.00067 ; PubMed Central PMCID: PMC6816049. 30652541 PMC6816049

[15] IBM. IBM Explorys solutions 2021 [cited 2021 October 27]. Available from: https://www.ibm.com/watson-health/about/explorys.

[16] Pisoni RL, Gillespie BW, Dickinson DM, Chen K, Kutner MH, Wolfe RA. The Dialysis Outcomes and Practice Patterns Study (DOPPS): design, data elements, and methodology. Am J Kidney Dis 2004;44:7–15. doi: 10.1053/j.ajkd.2004.08.005 . 15486868

[17] Herrett E, Gallagher AM, Bhaskaran K, Forbes H, Mathur R, van Staa T, et al. Data Resource Profile: Clinical Practice Research Datalink (CPRD). Int J Epidemiol 2015;44:827–36. doi: 10.1093/ije/dyv098 ; PubMed Central PMCID: PMC4521131. 26050254 PMC4521131

[18] Tanaka S, Seto K, Kawakami K. Pharmacoepidemiology in Japan: medical databases and research achievements. J Pharm Health Care Sci 2015;1:16. doi: 10.1186/s40780-015-0016-5 ; PubMed Central PMCID: PMC4729130. 26819727 PMC4729130

[19] Nakagawa N, Sofue T, Kanda E, Nagasu H, Matsushita K, Nangaku M, et al. J-CKD-DB: a nationwide multicentre electronic health record-based chronic kidney disease database in Japan. Sci Rep 2020;10:7351. doi: 10.1038/s41598-020-64123-z ; PubMed Central PMCID: PMC7192920. 32355258 PMC7192920

[20] Schneeweiss S, Brown JS, Bate A, Trifiro G, Bartels DB. Choosing Among Common Data Models for Real-World Data Analyses Fit for Making Decisions About the Effectiveness of Medical Products. Clin Pharmacol Ther 2020;107:827–33. doi: 10.1002/cpt.1577 . 31330042

[21] Young EW, Goodkin DA, Mapes DL, Port FK, Keen ML, Chen K, et al. The Dialysis Outcomes and Practice Patterns Study (DOPPS): An international hemodialysis study. Kidney Int 2000;57:S74–S81. doi: 10.1046/j.1523-1755.2000.07413.x

[22] Klann JG, Abend A, Raghavan VA, Mandl KD, Murphy SN. Data interchange using i2b2. J Am Med Inform Assoc 2016;23:909–15. doi: 10.1093/jamia/ocv188 ; PubMed Central PMCID: PMC4997035. 26911824 PMC4997035

[23] Overhage JM, Ryan PB, Reich CG, Hartzema AG, Stang PE. Validation of a common data model for active safety surveillance research. J Am Med Inform Assoc 2012;19:54–60. doi: 10.1136/amiajnl-2011-000376 ; PubMed Central PMCID: PMC3240764. 22037893 PMC3240764

[24] Curtis LH, Weiner MG, Boudreau DM, Cooper WO, Daniel GW, Nair VP, et al. Design considerations, architecture, and use of the Mini-Sentinel distributed data system. Pharmacoepidemiol Drug Saf 2012;21 Suppl 1:23–31. doi: 10.1002/pds.2336 . 22262590

[25] Ball R, Robb M, Anderson SA, Dal Pan G. The FDA’s sentinel initiative—A comprehensive approach to medical product surveillance. Clin Pharmacol Ther 2016;99:265–8. doi: 10.1002/cpt.320 . 26667601

[26] PCORnet. PCORnet Common Data Model (CDM) Specification, Version 5.1 2019 [cited 2020 December 22]. Available from: https://pcornet.org/wp-content/uploads/2019/09/PCORnet-Common-Data-Model-v51-2019_09_12.pdf.

[27] Weeks J, Pardee R. Learning to Share Health Care Data: A Brief Timeline of Influential Common Data Models and Distributed Health Data Networks in U.S. Health Care Research. EGEMS (Wash DC) 2019;7:4. doi: 10.5334/egems.279 ; PubMed Central PMCID: PMC6437693. 30937326 PMC6437693

[28] ScyMed Inc. MediCalc Conversion 2019 [cited 2020 December 22]. Available from: http://www.scymed.com/en/smnxfd/smnxfdam.htm.

[29] UnitsLab.com. The resource for conversion SI units to conventional or traditional units used in laboratory and medical practice 2020 [cited 2020 December 22]. Available from: http://unitslab.com/.

[30] Levey AS, Stevens LA, Schmid CH, Zhang YL, Castro AF, 3rd, Feldman HI, et al. A new equation to estimate glomerular filtration rate. Ann Intern Med 2009;150:604–12. doi: 10.7326/0003-4819-150-9-200905050-00006 ; PubMed Central PMCID: PMC2763564. 19414839 PMC2763564

[31] Matsuo S, Imai E, Horio M, Yasuda Y, Tomita K, Nitta K, et al. Revised equations for estimated GFR from serum creatinine in Japan. Am J Kidney Dis 2009;53:982–92. doi: 10.1053/j.ajkd.2008.12.034 . 19339088

[32] Papermill. Welcome to papermill 2018 [cited 2021 July 5]. Available from: https://papermill.readthedocs.io/en/latest/index.html.

[33] Wang SV, Schneeweiss S, Berger ML, Brown J, de Vries F, Douglas I, et al. Reporting to Improve Reproducibility and Facilitate Validity Assessment for Healthcare Database Studies V1.0. Value Health 2017;20:1009–22. doi: 10.1016/j.jval.2017.08.3018 . 28964431

[34] Rijnbeek PR. Converting to a common data model: what is lost in translation?: Commentary on "fidelity assessment of a clinical practice research datalink conversion to the OMOP common data model". Drug Saf 2014;37:893–6. doi: 10.1007/s40264-014-0221-4 . 25187018

[35] Xu H, Ashfaq A, Karaboyas A, Tentori F, Jadoul M, Locatelli F, et al. Prevalence of Hyperkalemia in Dopps: A Real-World, International Cohort of Hemodialysis Patients. Nephrol Dial Transplant 2017;32:iii563–iii. doi: 10.1093/ndt/gfx170.MP371

[36] Cohen AT, Goto S, Schreiber K, Torp-Pedersen C. Why do we need observational studies of everyday patients in the real-life setting? European Heart Journal Supplements 2015;17:D2–D8. doi: 10.1093/eurheartj/suv035

[37] James G, Nyman E., Fitz-Randolph M., Niklasson A., Hedman K., Hedberg J., et al. Characteristics, Symptom Severity, and Experiences of Patients Reporting Chronic Kidney Disease in the PatientsLikeMe Online Health Community: Retrospective and Qualitative Study. J Med Internet Res 2020;22:e18548. doi: 10.2196/18548 ; PubMed Central PMCID: PMC7391670. 32673242 PMC7391670

[38] James GJ-JC, Supriya Kumar, Steven Fishbane, Carol Moreno Quinn, Eric Wittbrodt, Eiichiro Kanda, et al. PO1461: Characteristics of chronic kidney disease patients with hyperkalemia: A report from the DISCOVER CKD retrospective cohort. 2020.

[39] Center for Devices and Radiological Health. Use of Real-World Evidence to Support Regulatory Decision-Making for Medical Devices 2017 [cited 2020 November 10]. Available from: https://www.fda.gov/regulatory-information/search-fda-guidance-documents/use-real-world-evidence-support-regulatory-decision-making-medical-devices.

[40] Medicines & Healthcare products Regulatory Agency. Consultation document: MHRA draft guidance on randomised controlled trials generating real-world evidence to support regulatory decisions 2020 [cited 2021 February 03]. Available from: https://www.gov.uk/government/consultations/mhra-draft-guidance-on-randomised-controlled-trials-generating-real-world-evidence-to-support-regulatory-decisions/consultation-document-mhra-draft-guidance-on-randomised-controlled-trials-generating-real-world-evidence-to-support-regulatory-decisions.

[41] Kent S, Burn E, Dawoud D, Jonsson P, Ostby JT, Hughes N, et al. Common Problems, Common Data Model Solutions: Evidence Generation for Health Technology Assessment. Pharmacoeconomics 2021;39:275–85. doi: 10.1007/s40273-020-00981-9 ; PubMed Central PMCID: PMC7746423. 33336320 PMC7746423

