# Supplementary material for: Developing a common data model approach for DISCOVER CKD: A retrospective, global cohort of real-world patients with chronic kidney disease
Source: PLoS One. 2022 Sep 29;17(9):e0274131. doi: 10.1371/journal.pone.0274131 (PMC9521926; doi:10.1371/journal.pone.0274131)
Supplement: S1 Table — (DOCX) [file pone.0274131.s002.docx]

S1 Table. Target Data Standards for DISCOVER CKD

| **Data Element Category** | **Coding System** |
| --- | --- |
| Diagnosis | ICD-9, ICD-10, Read code, CPRD medcode, MDV disease code |
| Treatment | CPRD product codes, NDC, RxNorm, ATC, ATC MDV, MDV receipt code |
| Laboratory measures | Read code, CPRD medcode, MDV lab, LOINC, CPRD enttype |
| Procedures | Read code, CPRD medcode, ICD-9, ICD-10, CPT-4, HCPCS, MDV receipt code, OPCS |

ATC, Anatomical Therapeutic Chemical code; CKD, chronic kidney disease; CPRD, Clinical Practice Research Datalink; CPT-4, Current Procedural Terminology, 4th Edition; HCPCS, Healthcare Common Procedure Coding System; ICD9, International Classification of Diseases, Ninth Revision; ICD10, International Classification of Diseases, Tenth Revision; LOINC, Logical Observation Identifiers Names and Codes; MDV, Medical Data Vision; NDC, National Drug Code; OPCS, Office of Population Censuses and Surveys.
