# Supplementary material for: Developing a common data model approach for DISCOVER CKD: A retrospective, global cohort of real-world patients with chronic kidney disease
Source: PLoS One. 2022 Sep 29;17(9):e0274131. doi: 10.1371/journal.pone.0274131 (PMC9521926; doi:10.1371/journal.pone.0274131)
Supplement: S2 Table — (DOCX) [file pone.0274131.s003.docx]

S2 Table. Study Team Members, Roles, and Affiliations

| Name | Role | Affiliation |
| --- | --- | --- |
| Glen James | Epidemiologist/Scientific Lead | Formerly AstraZeneca |
| Eric Wittbrodt | Epidemiologist | AstraZeneca |
| Alyshah Abdul Sultan | Epidemiologist | Formerly AstraZeneca |
| Supriya Kumar | Data Scientist Lead | AstraZeneca |
| Matthew Arnold | Data Scientist | AstraZeneca |
| Tony Chen | Statistical Lead | AstraZeneca |
| Juan Jose Garcia Sanchez | Payer Evidence Lead | AstraZeneca |
